# Management of Vertebral Defects, Anal Atresia, Cardiac Defects, Tracheoesophageal Fistula or Atresia, Renal Anomalies, and Limb Abnormalities (VACTERL) in a Child With Complex Medical Needs in the Foster Care System: A Pediatric Case Study

**DOI:** 10.7759/cureus.65581

**Published:** 2024-07-28

**Authors:** Arianna R Gregg, Bertha A Beltran-Regalado, Mackenzie L Montero, Roshan M Panda, Jose Cucalon Calderon

**Affiliations:** 1 Pediatrics, University of Nevada Reno School of Medicine, Reno, USA

**Keywords:** pediatrics, multi-disciplinary care, foster care, complex medical needs, vacterl association

## Abstract

Vertebral defects, anal atresia, cardiac defects, tracheoesophageal fistula or atresia, renal anomalies, and limb abnormalities (VACTERL) association is a complex congenital condition characterized by the presence of malformations that affect various organ systems. Most children born with VACTERL association require surgery shortly after birth, often undergoing multiple procedures during infancy, which can lead to a wide range of physical challenges. The unique combination of malformations in these children in addition to having complex care needs that need to be met can result in physical and social difficulties in their daily lives, affecting both their own and their caregivers' quality of life.

In some cases, children with complex medical needs are placed in foster care. When children with complex health needs enter the foster care system, there is a risk of overwhelming the caretaker, leading to their needs continuing to be unmet. Pediatricians have a role not only in helping support families but also in knowing what resources are available to meet these needs, which can be dependent on what their communities offer. Pediatricians require current training to navigate their state's foster care system. This training allows pediatricians to effectively collaborate with foster families while also assisting and coordinating complex care to support these families. We present a case of a child with complex health needs placed in the foster care system, facing multiple healthcare challenges, with care delayed due to difficulty attending appointments. Highlighted is the importance of delivering supportive, personalized, and multidisciplinary care to families with children who have complex health needs, including when caretakers are within the foster care system.

## Introduction

VACTERL is an acronym that encompasses a wide range of congenital abnormalities and is clinically characterized by vertebral, anorectal, cardiac, renal, and limb anomalies, as well as tracheoesophageal fistula or atresia [1]. To improve survival, many children diagnosed with VACTERL association undergo surgery shortly after birth, often followed by multiple procedures and follow-ups with specialists throughout infancy. This medical journey can lead to a diverse array of physical challenges [2]. Children with complex medical needs are often placed in foster care, a system that annually supports almost 700,000 children in the United States facing temporary or permanent separation from their family of origin [3]. It is estimated that approximately 20,000-40,000 children annually, constituting 5-10% of the total foster care population, are placed in foster care due to a complex medical history [4].

In 2018, a staggering 680,000 children experienced time in foster care, predominantly attributed to neglect (62%), parental substance abuse (36%), challenges in coping with a parental role (14%), or physical abuse (13%) [3]. For children with intricate medical needs necessitating intensive care or supervision, foster care arrangements may extend to residential group care settings. While these settings offer healthcare and supervision, they can also contribute to pediatric health disparities, influenced by factors such as poverty, single-parent households, maternal mental health issues, and instances of community and household violence [4]. 

Here, we report a case of a premature infant with a complex birth history. The infant’s delivery and post-partum clinical course were complicated by respiratory distress syndrome, syphilis exposure, and intrauterine drug exposure to amphetamine, methamphetamine, norbuprenorphine, and tetrahydrocannabinol. After an evaluation of physical features, congenital anomalies were noted and the infant was diagnosed with VACTERL. Once medical clearance was obtained, the neonate was placed under the sole care of a foster parent. This case highlights a child with complex care needs in foster care and the ongoing need to support foster parents in meeting the child's needs and ensuring their overall well-being. 

## Case presentation

Our patient was a premature infant with a complex birth history. This patient’s birth was complicated by respiratory distress syndrome, syphilis exposure, and intrauterine drug exposure to amphetamine, methamphetamine, norbuprenorphine, and tetrahydrocannabinol. ​​The infant was delivered prematurely at 35 weeks gestation. Since birth, the infant was placed in various critical care settings for further care and evaluation once multiple congenital anomalies were noted. On day nine of life, the infant was admitted to the Pediatric ICU (PICU) for ongoing management of imperforate anus with anal dilations and feeding management with a combination of oral and nasogastric feeding tubes.

Before discharge, the patient underwent an ophthalmology exam for secondary syphilis exposure and a repeat kidney, ureters, and bladder (KUB) X-ray to verify a bubbly bowel gas pattern, previously thought to be stools. The patient also needed to complete her antibiotic regimen before discharge due to her syphilis exposure. During her time in critical care, she was monitored for opioid withdrawal. Furthermore, Child Protective Services (CPS), whose involvement was necessary due to a history of drug use during the pregnancy, had not yet cleared the patient to go home. CPS stated that they had been unable to reach the mother and that there was not enough information to relinquish custody from the mother at that point in time. Communication was further challenged as the mother reported being homeless. On day 11 of life, the mother came to the bedside but struggled to comprehend the situation of her child. Her speech was slurred and her gait was unbalanced.

**Figure 1:**
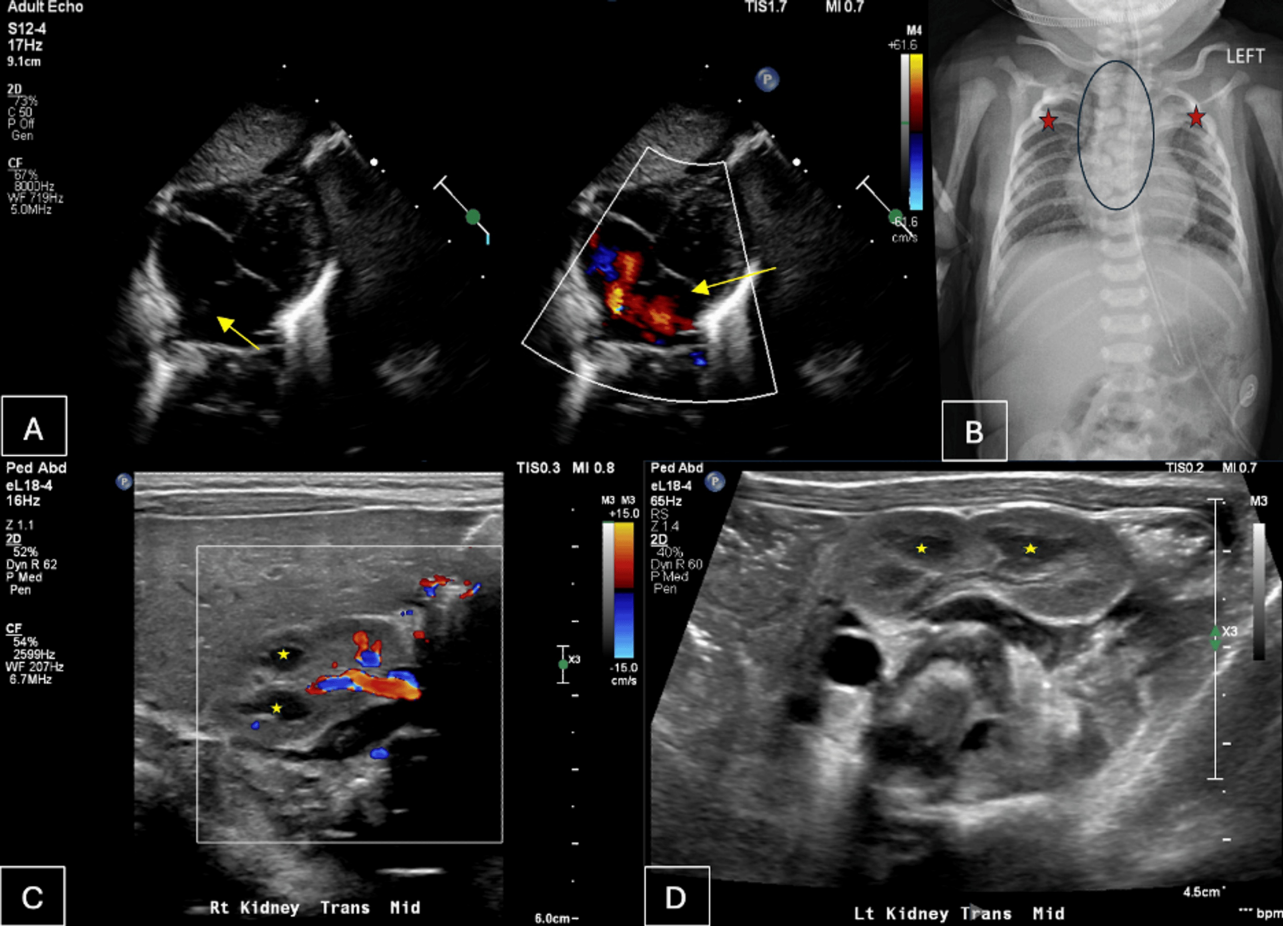
VACTERL imaging findings (A) Two-dimensional (2D) echocardiography of the patient showing a small to moderate-sized secundum type atrial septal defect (yellow arrow) with left to right shunt noted by the red-colored flow across the atrial septal defect (ASD). (B) Frontal view chest X-ray of the patient showing multiple thoracic vertebral anomalies (black circle) in addition to distortion of ribs bilaterally (red asterisks). Pediatric abdominal ultrasound of the patient with a transverse view of the (C) right kidney and (D) left kidney showing bilateral renal calyceal dilation (yellow asterisks) without significant dilation of the renal pelvis, most consistent with congenital megacalyces. Hilar vessels are indicated by the color Doppler (C). ASD, atrial septal defect; VACTERL: vertebral defects, anal atresia, cardiac defects, tracheoesophageal fistula or atresia, renal anomalies, and limb abnormalities

Further workup showed a poorly feeding infant with a low imperforate anus treated with serial dilations, an echocardiogram (echo) showed a moderate atrial septal defect, bilateral renal calyceal dilation was noted on renal ultrasound (US), multiple thoracic vertebral and rib anomalies were seen on X-ray, and the patient tested positive for alpha thalassemia trait (Figure 1). The patient had adequate stool output following the dilation procedures. A neonatal eye exam was unremarkable. Based on the patient’s presentation, referrals to ophthalmology, pediatric surgery, cardiology, nephrology, urology, orthopedics, and hematology and oncology were placed to provide adequate care for the patient. Upon medical clearance from the hospital, the patient was discharged into foster care on day 16 of life.

The neonate returned with her foster parent to her primary care provider (PCP) at three weeks of age. Upon further evaluation, the infant was found to have a wide range of congenital abnormalities and was subsequently diagnosed with VACTERL (vertebral, anorectal, cardiac, and renal anomalies). She was found to be gaining weight and growing with normal development. During this visit, the child met appropriate milestones and her PCP assisted in coordinating the necessary appointments based on the referrals for continued care. Following this visit, an appointment was made with urology; however, the patient did not show up for this appointment. An attempt to reschedule was made but was unsuccessful with no reason listed. The patient’s foster parent utilized online communication platforms to discuss medical concerns such as rashes with the primary care team in an attempt to minimize additional in-person appointments. Three months after delivery, the infant underwent a posterior sagittal anorectoplasty without complaint to correct the infant’s imperforate bowel.

During this hospital stay, the biological mother of the child attempted to visit the infant while suspected to be under the influence of alcohol due to both behavioral and motor cues. The hospital staff coordinated with both social work and hospital security to ensure the safety of the child and hospital staff. Throughout this stay, the patient was accompanied by a new social worker daily making communication between the patient’s care team immensely important. Five months after the infant’s birth the patient had been seen by four of the seven specialists that were consulted at birth, including pediatric nephrology, pediatric orthopedics, ophthalmology, and hematology and oncology. The infant’s PCP was instrumental in facilitating the continuation of care and served as an intermediary between specialists within and outside the hospital.

We can speculate that challenges regarding follow-up care first arose because of the discontinuity in care and the lack of effective communication. For the infant's first two weeks of life, she remained in her mother's custody, with all medical decisions being made by her. However, this was complicated by the mother being unreachable at times. Upon discharge, the infant was placed in a foster home with three additional foster children, which led to additional challenges. After reviewing the infant's medical record, it became clear that coordinating follow-up visits and appointments was challenging, with many appointments either being canceled or resulting in no-shows. Ultimately, the infant’s foster parent relied on online communication platforms to minimize additional in-person appointments. It is likely that, due to these reasons, the infant had not been seen by all the specialists who were consulted at birth till the last follow-up.

## Discussion

**Figure 2:**
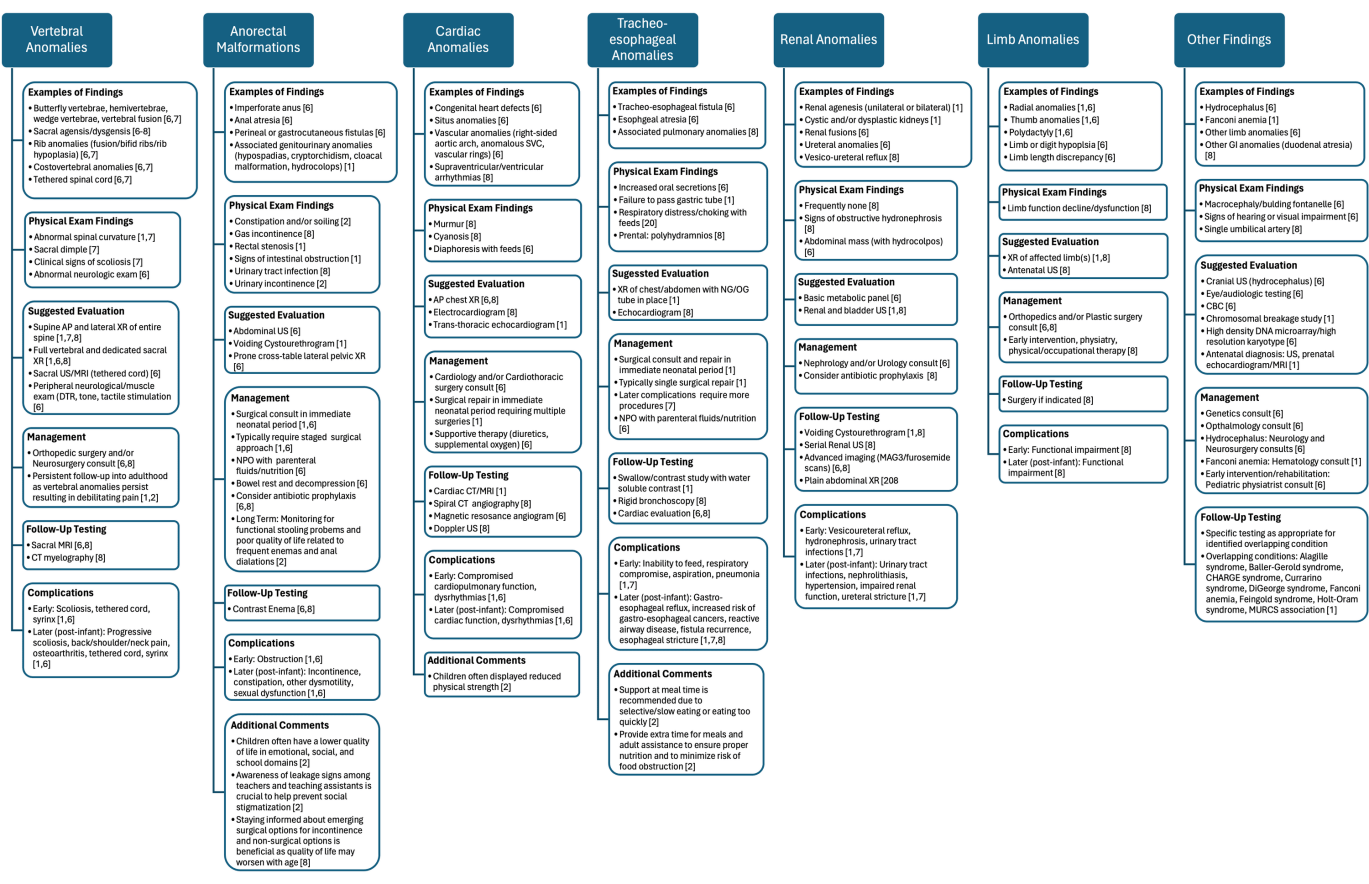
Assessment, treatment, and management of identified or suspected VACTERL association Graphical illustration of the proposed algorithm for neonates with VACTERL association and their associated sequelae of malformations [1,2,6,7,8]. AP, Anterior-Posterior; XR, X-ray; US, ultrasound; MRI, magnetic resonance imaging; DTR, deep tendon reflexes; NPO, nil per os (nothing by mouth); SVC, superior vena cava; CT, computed tomography; NG, nasogastric; OG, orogastric; CBC, complete blood count; MURCS, Müllerian duct aplasia, renal aplasia, and cervicothoracic somite dysplasia Image Credit: The authors of this article

This patient with a diagnosis of VACTERL required complex medical care and evaluation by multiple specialists to adequately treat the patient’s imperforate anus as well as prevent further complications due to her cardiac, renal, and vertebral anomalies. Due to the complexity of this disease, timely follow-up and medical care are crucial to ensure a positive outcome in children with this disease yet there is no standard approach for the initial diagnostic work-up [1]. This may lead to missed manifestations, which can complicate the etiological workup, delay medical interventions, potentially contributing to higher morbidity and mortality, and ultimately result in less effective and informed counseling [1]. We have developed an algorithm that we believe will be valuable for a broad range of medical professionals. Although the recommended diagnostic process may involve significant costs, applying this algorithm in early infancy could ultimately reduce both morbidity rates and overall healthcare expenses (Figure 2). Previous literature has identified specific barriers such as inadequate insurance, poor health literacy, or misdiagnosis by the physician as reasons for poor outcomes and follow-up rates [5]. This case highlights the intricate nature of coordinating care for patients navigating social factors like being in foster care. It underscores the vital importance of clinicians being attuned to the challenges patients face in accessing specialized care for complex conditions. 

Caring for children with complex medical needs is often akin to outpatient intensive care, demanding significant resources and meticulous attention [9]. Despite income, language, or insurance status, families tending to such children frequently encounter unmet healthcare needs [4]. Limited data on medical foster families suggests that many foster parents feel ill-prepared for the challenges of caring for children with complex medical conditions [10]. For instance, in children who have undergone posterior sagittal anorectoplasty, as seen in our patient, it is recommended to perform anal dilatations twice daily [11]. Children with complex medical needs often experience multiple foster home placements, resulting in disrupted communication about their health issues [4].

Incomplete medical history of foster children adds to the difficulties faced by foster parents in navigating the healthcare system and accessing essential services [10]. Concerns persist that withheld medical information may discourage foster parents from accepting placements, contributing to a significant decline in licensed foster caregivers willing to care for medically complex children [12] and placing these caregivers at higher risk of poor health literacy and healthcare distrust. The complexity of VACTERL coupled with the enhanced challenges of coordinating care in foster care increases the need for clear communication and enhanced awareness by the healthcare team to ensure that adequate support is provided to maintain a positive healthcare relationship and deliver vital care to these children. Fortunately, our patient was able to undergo the necessary surgery and consult with multiple specialties in a delayed but still timely manner; however, it required immense communication and flexibility to reschedule the necessary appointments and frequent communication with her primary care pediatrician to help coordinate and support these needs. 

Decades of research have consistently shown that almost half of all children in foster care experience chronic medical issues, and up to 80% experience serious emotional struggles [13]. Despite this overwhelming evidence of need, studies consistently reveal that many healthcare needs of children in the foster care system remain unmet [13]. Inadequate training and preparation for foster parents can also contribute to these children's unmet healthcare needs [4]. One study revealed that approximately 35% of licensed foster care families in the United States lack the inclination or proficiency to care for children with special needs, resulting in a shortage of placements for foster children [14]. Due to these challenges, caregivers experience a significant burden [2].

**Figure 3:**
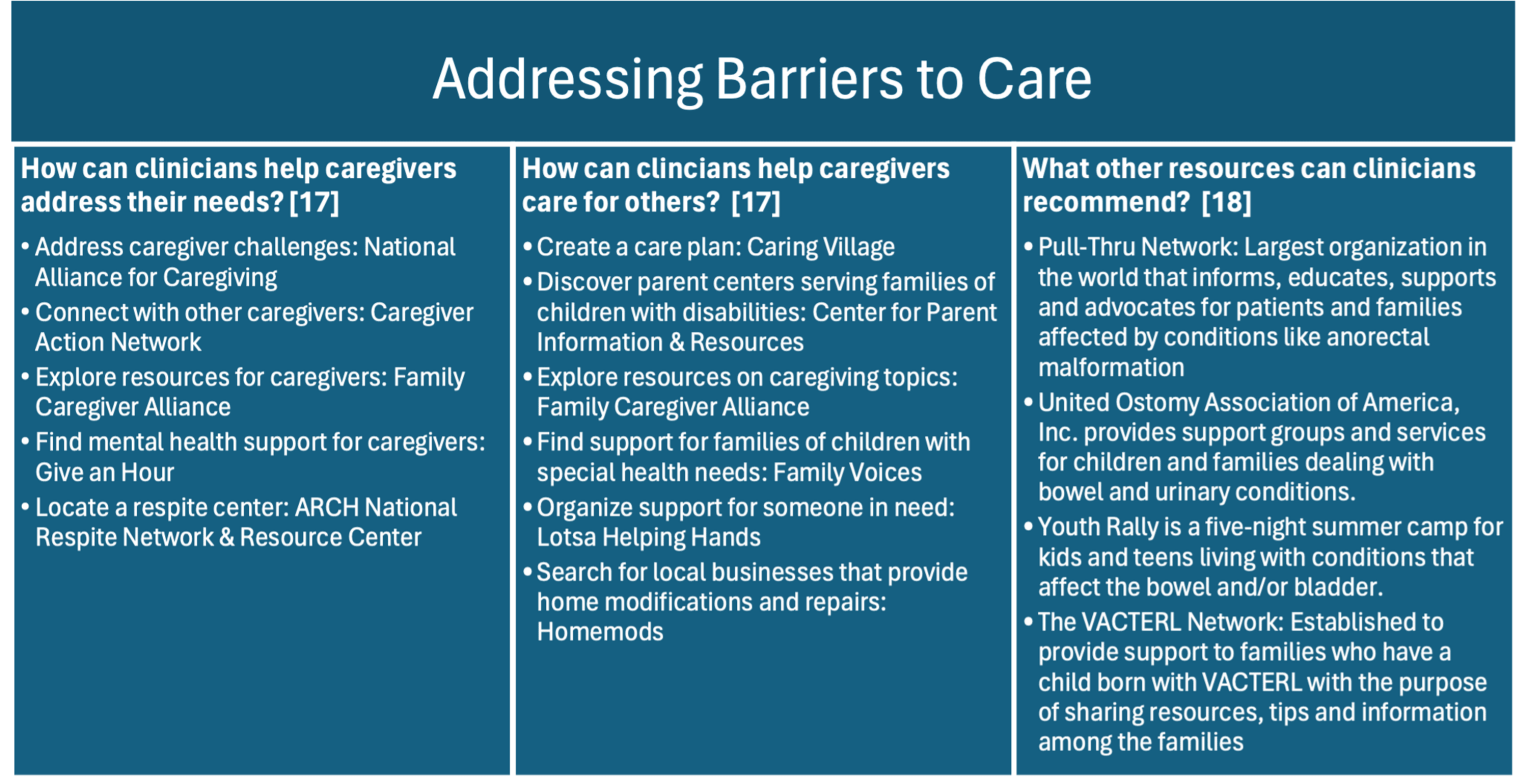
Support resources for caregivers: navigating challenges and finding community Examples of resources for those taking care of an individual with VACTERL association [17]. Format for the first two columns - topic addressed: resource/website ARCH: Access to Respite Care and Help; VACTERL: vertebral defects, anal atresia, cardiac defects, tracheoesophageal fistula or atresia, renal anomalies, and limb abnormalities Image Credit: The authors of this article

In a study conducted by Skreden et al., approximately 30% of parents of children with a malformation reported symptoms of clinically significant psychological distress and anxiety nine years after their child’s birth [15]. Additionally, previous studies involving parents of children with congenital malformations have reported higher scores in depression and anxiety, as well as increased psychological distress and more depressive symptoms compared to parents of children without congenital malformations [16]. To cope, caregivers require psychological support, quality medical care, guidance from experts, and peer support from other caregivers with a few of these resources highlighted in Figure 3. Developing a care plan alongside pediatricians that provides individualized and tailored support for each child, along with appropriate training and support for the caregivers, is essential [3]. This should include caretakers throughout the foster care system. 

Pediatricians in charge of medical care for these children bear these complex responsibilities, which encompass closing information gaps in both medical and social contexts, delivering comprehensive care, and acting as intermediaries between specialists within and outside the hospital system [4]. While caregivers may not initially recognize their need for psychosocial support, it's important to offer routine support [16]. Previous research has noted that caregivers often feel isolated and lack familiarity with other children and caregivers in similar situations [18]. Therefore, facilitating meetings with other children and their caregivers who have the same, or a similar, diagnosis could provide recognition, hope, and mutual support, and perhaps result in lasting relationships [18]. 

Additionally, primary clinicians hold crucial roles in contributing to medical decisions during frequent inpatient hospitalizations of these children. Motivated pediatricians who are willing to care for complex patients also need specialized training and access to resources to deliver the appropriate level of care [19]. In 2003, the Institute of Medicine recommended that all pediatric clinicians receive training to provide safe, efficient, effective, and equitable care to medically complex patients [19]. Nonetheless, this recommendation has not yet resulted in the widespread availability of continuing medical education [19]. It is imperative that all pediatric clinicians receive training on navigating their state's foster care system, remain empathetic to the additional challenges faced by these patients, and increase their flexibility to accommodate scheduling conflicts. Additionally, it is crucial for pediatric clinicians to be aware of what is available in their communities to support children with complex care needs and their caretakers. 

## Conclusions

This case of a premature infant with VACTERL association and complex medical needs in the foster care system underscores the profound challenges faced by both the children and their caregivers. It highlights the necessity for a multidisciplinary approach to ensure timely and coordinated medical care. The intricate nature of VACTERL, coupled with the complexities of the foster care system, necessitates clear communication, flexibility, and robust support mechanisms for caregivers. Despite the significant barriers, our patient's case demonstrates the potential for positive outcomes with dedicated and coordinated care. However, it also brings to light the critical need for enhanced training for pediatricians and foster caregivers to manage these complexities effectively. Comprehensive training, empathetic understanding, and awareness of community resources are essential to providing equitable and efficient care for medically complex children. Moving forward, it is imperative to address the systemic gaps in training and resources, ensuring that pediatric clinicians are well-equipped to handle the unique challenges faced by children with VACTERL in foster care. Additionally, fostering connections among caregivers and providing them with psychological and peer support can alleviate the immense burden they bear. By addressing these needs, we can improve the quality of life and health outcomes for these vulnerable children, ensuring that they receive the care and support they deserve.
